# Identifying species traits that predict vulnerability to climate change

**DOI:** 10.1017/ext.2024.24

**Published:** 2024-12-05

**Authors:** Damien A. Fordham

**Affiliations:** 1 The Environment Institute and School of Biological Sciences, University of Adelaide, Adelaide, SA 5005, Australia; 2 Center for Macroecology, Evolution and Climate, Globe Institute, University of Copenhagen, Copenhagen, Denmark; 3 Center for Mountain Biodiversity, Globe Institute, University of Copenhagen, Copenhagen, Denmark

**Keywords:** biodiversity conservation, extinction risk, process-based model, range shift, resurvey data

## Abstract

Accurately predicting the vulnerabilities of species to climate change requires a more detailed understanding of the functional and life-history traits that make some species more susceptible to declines and extinctions in shifting climates. This is because existing trait-based correlates of extinction risk from climate and environmental disturbances vary widely, often being idiosyncratic and context dependent. A powerful solution is to analyse the growing volume of biological data on changes in species ranges and abundances using process-explicit ecological models that run at fine temporal and spatial scales and across large geographical extents. These simulation-based approaches can unpack complex interactions between species’ traits and climate and other threats. This enables species-responses to climatic change to be contextualised and integrated into future biodiversity projections and to be used to formulate and assess conservation policy goals. By providing a more complete understanding of the traits and contexts that regulate different responses of species to climate change, these process-driven approaches are likely to result in more certain predictions of the species that are most vulnerable to climate change.

## Impact statement

I review different approaches for detecting extinction risk from climate change using species traits. This identified vital roles for process-explicit models in enriching knowledge of functional and life history traits that regulate species’ responses to shifting climates. More frequent application of these process-driven approaches to natural history and resurvey data will better identify species that are most vulnerable to climate change.

## Introduction

Anthropogenic climate change is already having measurable ecological impacts (Scheffers et al., 2016), and these impacts are set to intensify in the coming decades (Steffen et al., 2018). Consequently, accurate predictions of biodiversity responses to climate change are urgently needed to better guide conservation, and prevent wide-scale climate-driven biodiversity loss (Fordham et al., 2020). However, generating these predictions with a high level of confidence will require a much stronger understanding of the ecological and biophysical mechanisms that cause populations to decline, and species to go extinct in shifting climates (Briscoe et al., 2023; Urban et al., 2016).

Most predictions of species’ vulnerability to climate change do not directly consider biological processes (Urban, 2015), and this can affect their accuracy (Fordham et al., 2018). Ecological, demographic and biophysical mechanisms are commonly omitted from climate-biodiversity predictions due to insufficient knowledge and data on biotic responses to climate change (Pacifici et al., 2015). Another reason is that the ecological models needed to directly simulate these mechanistic responses are generally complex and computationally demanding, often requiring specific training and high-performance computing (Briscoe et al., 2019). Consequently, the proximate causes of climate-driven declines of species remain largely unclear (Moritz and Agudo, 2013).

Measurements of species functional traits (morphological, physiological and phenological characteristics) that capture variation in life history (growth, reproduction and survival) strategies (Box 1) have developed rapidly in recent decades (Kattge et al., 2020; Tobias et al., 2022; Wilman et al., 2014), along with standards for collecting and collating this data, ensuring greater interoperability (Edgar and Stuart-Smith, 2014). This information, which is now widely accessible through online databases, is being used to identify species traits associated with differences in timings and rates of recent range shifts (Bradshaw et al., 2014; Estrada et al., 2015; Tingley et al., 2012), and establish species and areas most vulnerable to future exposure to climate change (Andrew et al., 2022; Foden et al., 2013; Garcia et al., 2014a). However, the strength of inference that can be made from these (and other) inter-specific trait-based assessments, requires the processes affecting organismal performance in shifting climates and environments to be directly captured in the functional and life-history traits being analysed, which does not always happen (Beissinger and Riddell, 2021; Schleuning et al., 2020).

Box 1. Key definitions.

**Biophysical model:** physiological constraints of a species are used to model the environmental conditions under which it can exist.

**Functional traits**: morphological, physiological, biochemical and phenological characteristics expressed in the phenotypes of individual organisms, which regulate responses to their environment.

**Individual-based model:** directly captures how individuals interact and adapt to the system that the species is embedded in.

**Life-history traits:** Demographic characteristics of a species that affect its fitness, including age at maturity, population growth rate, survival rates, sex ratio etc.

**Pattern-oriented modelling:** an approach for using empirical patterns for the selection and calibration of models of complex systems.

**Population model:** a mathematical model that simulates species’ demographic rates to address both the structure and dynamics of its population.

**Process-explicit model:** causal based models that represent the dynamics of a system as explicit functions of the events that drive change in that system.

Recent developments in macroecological and biophysical modelling provide new opportunities to directly improve knowledge of the functional and life-history traits that render some species vulnerable to declines and extinctions. These mechanistic approaches are enabling intrinsic traits that regulate species’ responses to climate and other environmental change to be disentangled and deciphered (Tomlinson et al., 2024). Here, proposed mechanisms are encoded into process-explicit models that simulate movement, mortality and reproduction in networks of populations across time. The patterns that they produce are validated against real-world data or theoretical expectation, allowing complex interactions between ecological and behavioural processes, climatic drivers, and other threatening activities to be separated and dissected (Pilowsky et al., 2022a). By mechanistically linking species’ traits to organismal responses to climate and environmental disturbances, these process-driven simulation approaches can generate direct insights into the traits and ecological preferences of species that are likely to increase vulnerability to climate change (Canteri et al., 2022; Pearson et al., 2014). When implemented for multiple species, these process-explicit models provide opportunities to identify species at greatest risk of future population declines and extinction, encouraging pre-emptive rather than reactionary conservation efforts.

Here I examine approaches for predicting extinction risk from climate change using shared measurable characteristics of organisms. I outline why evidence for a relationship between species traits and extinction remains elusive, explaining why process-explicit models are needed to improve knowledge of the functional and life history traits that make some species more vulnerable to declines and extinction. Moreover, I describe how these approaches can be applied to natural history and resurvey data to better anticipate the potential ecological consequences of future climate change.

## Trait-based vulnerability to extinction

Evidence suggests that measurable characteristics of organisms—so called species’ biological traits—regulate the ecological processes of population declines and loss of species distributions (Chichorro et al., 2019; Kotiaho et al., 2005; Purvis et al., 2000). Ecological theory predicts that risks of extinction should be higher for animals with larger body sizes, smaller ranges, smaller population sizes and poorer dispersal abilities (McKinney, 1997; Pimm et al., 1988). Larger-bodied species tend to have slower life histories, smaller population densities and bigger home ranges, making them particularly susceptible to increased human-driven mortality (Ripple et al., 2017). Plants and animals with smaller ranges and population sizes are often more vulnerable to stochastic events (Matthies et al., 2004), while species with reduced dispersal capacities are often more prone to climate and environmental disturbances because they are unable to move large distances (Chichorro et al., 2022). Although these traits (supported by theory) are relatively easy to measure or derive, comparative analyses of extinction risk have shown that correlates of vulnerability to population declines and range contractions vary widely, often being idiosyncratic and context-dependent (Davidson et al., 2009; Fisher and Owens, 2004; Owens and Bennett, 2000).

Accordingly, empirical studies have yielded opposing explanations for biological mechanisms underlying patterns of extinction risk (Beissinger, 2000) and the central question of what ecological characteristics make certain species more vulnerable to population declines and extinctions has never been fully resolved. What is clear, however, is that the ecological mechanisms that put species at risk of extinction are complex, and that they vary depending on the threatening process or combination of processes, including habitat loss, overexploitation and invasive species (González-Suárez et al., 2013; Owens and Bennett, 2000). This makes predicting ecological outcomes (e.g., population declines, distributional shifts) from species traits difficult, particularly since threatening processes tend to operate in unison, often having synergistic effects (Brook et al., 2008).

## Linking species traits to climate change responses

While it is estimated that one in six species is at risk of extinction from human-driven climate change (Urban, 2015), the proximate causes of observed population declines and range contractions in shifting climates remain poorly understood. Consequently, knowledge of how species traits mediate climate-driven changes in the abundances and distributional boundaries of plants and animals is unclear (Angert et al., 2011; Beissinger and Riddell, 2021; Cahill et al., 2013). The reasons include a general lack of information on functional and life-history responses to climate change for most species (Garcia et al., 2014b), and the mainly statistical methods being used to analyse the scarce amount of relevant data that is available (Green et al., 2022).

Most predictions of vulnerability to climate change are done correlatively—not mechanistically—mainly using statistical-based ecological niche modelling approaches (Elith and Leathwick, 2009). Here, statistical relationships between aspects of climate and species’ occurrences are used to estimate the climatic conditions needed to maintain viable populations of a species today and how these may shift in the future (Araujo and Peterson, 2012). Modest data requirements, and simplifying assumptions, enable the potential impact of future climatic change on the distributions of thousands of species to be explored efficiently (Warren et al., 2018), providing useful statistical tools for forecasting impacts and designing conservation interventions (Guisan et al., 2013). Spatial projections of climate change exposure from ecological niche models have also been used to match species’ traits to responses to shifting climates (Garcia et al., 2014a). However, the capacity of niche modelling approaches to directly establish species traits that increase extinction risk from future climate change is more limited (Fordham et al., 2013a). This is because they predict potential exposure to climate change—based on the availability of climatically suitable habitat—not extinction risk, with no direct limitations imposed by species traits. Although, new approaches, which enable phenotypic plasticity and local adaptation of fitness-related traits to be accounted for in ecological niche models, are enabling climate-survival responses to be better captured in projections of vulnerability to climate change (Benito Garzón et al., 2019).

An alternative way to establish trait-based vulnerabilities of species to future climate change is to quantify their past effects on local extinctions, and distributional shifts using statistical models (Pacifici et al., 2015) (Table 1). Numerous studies have now used natural history collections and repeated surveys to statistically relate documented shifts in species geographic ranges to recent climatic change (Bradshaw et al., 2014; Estrada et al., 2015; Moritz et al., 2008; Tingley et al., 2012). When analysed together, using meta-analysis techniques, these studies of species range shifts can be linked to inter-specific variation in traits, allowing ecological function to be integrated into assessments of species’ vulnerability to climate change, and resultant conservation priority schemes (MacLean and Beissinger, 2017). This is done by asking, What are the ecological implications of species functional and life-history traits in changing climates for the fitness and persistence of species?

**Table 1.** Modelling approaches for identifying biological traits that predict vulnerability to climate change

Approach	Life-history traits	Functional traits	Causation	Spatially explicit	Large extents	Multiple interacting drivers
Statistical	X	X	.	X	X	.
Experimental	X	X	X	.	.	X
Biophysical	X	X	X	X	X	.
Individual-based	X	X	X	X	.	X
Population-based	X	.	X	X	X	X

Potential modelling approaches are statistical, experimental, biophysical, individual-based or population-based. Modelling approach determines capability to detect important functional as well as life-history traits, and establish whether links to climate shifts are based on causation or not. Most approaches are spatially explicit, while some approaches can disentangle trait-based responses at large (geographical) extents and in response to multiple (extrinsic) drivers of extinction risk that often interact synergistically. Note that “x” indicates that a function is present.

While these meta-analyses have detected negative impacts of human-induced climatic change on population declines and range shifts (Lenoir et al., 2020; Poloczanska et al., 2013), they have not been able to consistently link variation in observed responses to climatic change to different functional and life history traits (Angert et al., 2011; Beissinger and Riddell, 2021; MacLean and Beissinger, 2017). This is probably because of a combination of (i) methodical issues when quantifying population declines and range shifts (Beissinger and Riddell, 2021); (ii) difficulties in obtaining precise measurements of a wide enough spectrum of species traits (Schleuning et al., 2020); and (iii) problems with disentangling complex non-linear trait-based responses to climatic change, and their interplay with other anthropogenic impacts (Taheri et al., 2021).

Estimates of range shifts and changes in population trajectories generally have inherent uncertainties owing to imperfect detection and longitudinal changes in sampling effort and methods, which makes trait-based inferences often challenging (Hébert and Gravel, 2023). Moreover, commonly available functional and life history traits used in attribution studies of changes in distribution and abundance—namely, body size, fecundity and habitat breadth—are likely to only partially account for processes underpinning observed responses of species to climatic change (Schleuning et al., 2020). These issues, and a tendency for studies to use analytical techniques that assume linear relationships between species traits and demographic changes (Beissinger and Riddell, 2021), have meant that the biological traits of species identified as predictors of range shifts and local extinctions under climate change often differ widely between studies (Wheatley et al., 2017). This is seen even for assemblages of similar species in comparable ecosystems (Pinsky et al., 2013; Sunday et al., 2015). Another important issue is that climatic changes over attribution time periods are generally small compared to the effects of other human pressures (habitat loss and degradation, over exploitation etc.), making it difficult to confidently pinpoint species’ traits associated with climate vulnerability (Fordham et al., 2016).

Alternatively, small-scale field experiments, laboratory microcosm and larger-scale mesocosm experiments allow direct testing of the importance of variation in functional and life history traits in fluctuating environments, potentially improving knowledge of how future climate change will affect species’ persistence, the structure and function of ecological communities, and the food webs that they are embedded in (Stewart et al., 2013). They do this by providing tractable yet ecologically realistic bridges between simplified experimental conditions and the real world (Fordham, 2015). These experimental approaches indicate that trophic position, behaviour and life-history characteristics can all influence responses to climatic warming (Bestion et al., 2015; Ullah et al., 2018; Yvon-Durocher et al., 2015). While experiments can provide valuable information on trait-based responses to climate change that cannot be readily quantified from field-based surveys (Ullah et al., 2024), their insights are most relevant to the climate and environmental conditions under which the study was conducted, which can be vastly different to local and regional future conditions (De Boeck et al., 2015). Moreover, because experiments are difficult and expensive to construct and maintain, experimental inferences of climate change responses and vulnerabilities are available for only a narrow range of largely short-lived species, based on experiments that unavoidably simplify climatic forcing (Fordham, 2015).

While growth in experimental and observation data of species responses to climate change—and their synthesis using metanalysis—will undoubtably improve the taxonomic, spatial and temporal scale of trait-based vulnerability assessments, process-explicit simulation models provide an alternative and promising methodology for directly identifying ecological mechanisms of extinction and generating trait-based predictions of climate change vulnerability (Green et al., 2022). By generating spatially and temporally explicit projections of range and abundance, these mechanistic approaches enable important interactions between spatiotemporal drivers and demographic, physiological and behavioural responses to be disentangled (Pilowsky et al., 2022a).

## Process-explicit models

Species attributes that increase their risk of extinction from climate change can be dissected using process-driven models that simulate complex interplay between extrinsic (climatic and environmental) drivers and intrinsic (demographic, biophysical and behavioural) factors. For example, by simulating movement, mortality and reproduction in networks of populations across time, spatially- and temporally-explicit population models enable demographic responses to multiple and interacting anthropogenic impacts to be established (Fordham et al., 2013b). These process-explicit approaches have shown that vulnerability to extinction from future climate change can be predicted using life-history traits (e.g., generation length, growth rate, natal dispersal distance) and other ecological characteristics (e.g., range size, population size, niche breadth) that affect organismal performance (Pearson et al., 2014). Likewise, biophysical models that capture the exchange of energy and mass between an organism and its environment have identified functional traits (e.g., body surface area, basal metabolism, fur depth and density) and behavioural mechanisms (e.g., dispersal time, habitat use) that are likely to regulate species vulnerability to future climate change (Briscoe et al., 2022; Kearney et al., 2021). In biophysical approaches, the connection between trait and function is explicit. However, for population-based approaches the link is made without direct connections to species’ morphology, physiology, or behaviour (Table 1).

Biophysical models are particularly suited to providing robust predictions of range limits in new climates (Briscoe et al., 2019), and are now often used to establish functional traits that are likely to affect the distributional movements of species under future climate change (Briscoe et al., 2016; Kearney et al., 2009; Levy et al., 2015). These include the effects of shape, size, surface area and insulation on thermal tolerance (Kearney et al., 2021), and the importance of different life stages for fitness (Levy et al., 2015). Alternatively, spatially and temporally explicit population models provide computational frameworks that simulate geographic and demographic declines from multiple and interacting environmental threats (Fordham et al., 2018). This makes them particularly suitable for establishing species attributes that increase risk of population decline and extinction from multiple drivers of global change (Green et al., 2022). Individual-based modelling approaches are, in theory, better suited to identifying biological traits associated with vulnerability to climate change, because they simulate variation in trait values at the individual level (DeAngelis and Mooij, 2005). However, these approaches are computationally demanding, making them challenging to run at large geographic extents (Table 1). Nevertheless, they have established the importance of life-history and behavioural traits (particularly those mediating dispersal) in response to climate and environmental disturbance (Bocedi et al., 2014b).

## Pattern-oriented detection of traits

A scarcity of ecological data on species’ responses to climate change has long been viewed as a barrier to a wider application of biophysical, population- and individual-based models in biodiversity and climate change research, including trait-based analyses (Urban et al., 2016).

Pattern-oriented modelling uses observations as filters for evaluating whether an ecological model is adequate in its structure and parameterisation to simulate biological processes (Pilowsky et al., 2022b). Shifts in species geographic ranges and abundances are often used as targets in pattern-oriented analysis, enabling population-based, individual-based, and biophysical models to be built and optimised using parameters with wide but plausible ranges (Fordham et al., 2022; Grimm et al., 2005; Strubbe et al., 2023), including for data depauperate, vulnerable species (Pilowsky et al., 2023). These studies, using process-explicit models and pattern oriented methods, have revealed the importance of life-history traits in shifting climates and environments, including dispersal distance, population growth rate and allee effect (Fordham et al., 2024; Pilowsky et al., 2023).

Most recently, pattern-oriented modelling has emerged as a powerful tool for mechanistically reconstructing species’ demographic responses to multiple millennia of climate and environmental change, including periods when Earth’s climate warmed at rates similar to what is being forecast for the 21^st^ century (Fordham et al., 2020). This is being done using inferences of demographic and distributional change from fossils and ancient DNA (aDNA). These inferences are utilised as independent validation targets for identifying whether models have the structural complexity and parameterization needed to simulate species’ range shifts and population declines that happened hundreds to thousands of years ago (Pilowsky et al., 2022b). This multi-disciplinary approach—integrating the fields of macroecology, paleoecology, climatology, and genomics—is revealing species attributes that increase extinction risk during periods of rapid climatic change (Canteri et al., 2022). Applying this general framework to a greater diversity of taxa and time frames, including more recent observations of species range movements and population changes, will likely provide a more thorough understanding of the demographic traits that regulate species’ responses to climate change, leading to better predictions of the species that are most vulnerable to future climate change (Fordham et al., 2020).

## Looking ahead –

Climatologists have for decades been using process-explicit models and past observations to disentangle mechanisms of long-term climate forcing (Braconnot et al., 2012; Sherwood et al., 2020). Accordingly, a wider usage of process-explicit models for quantifying and reporting climate-driven biodiversity dynamics is likely to uncover the functional and life history traits that make some species more prone to climate change, improving conservation management and policy.

**Figure 1.**
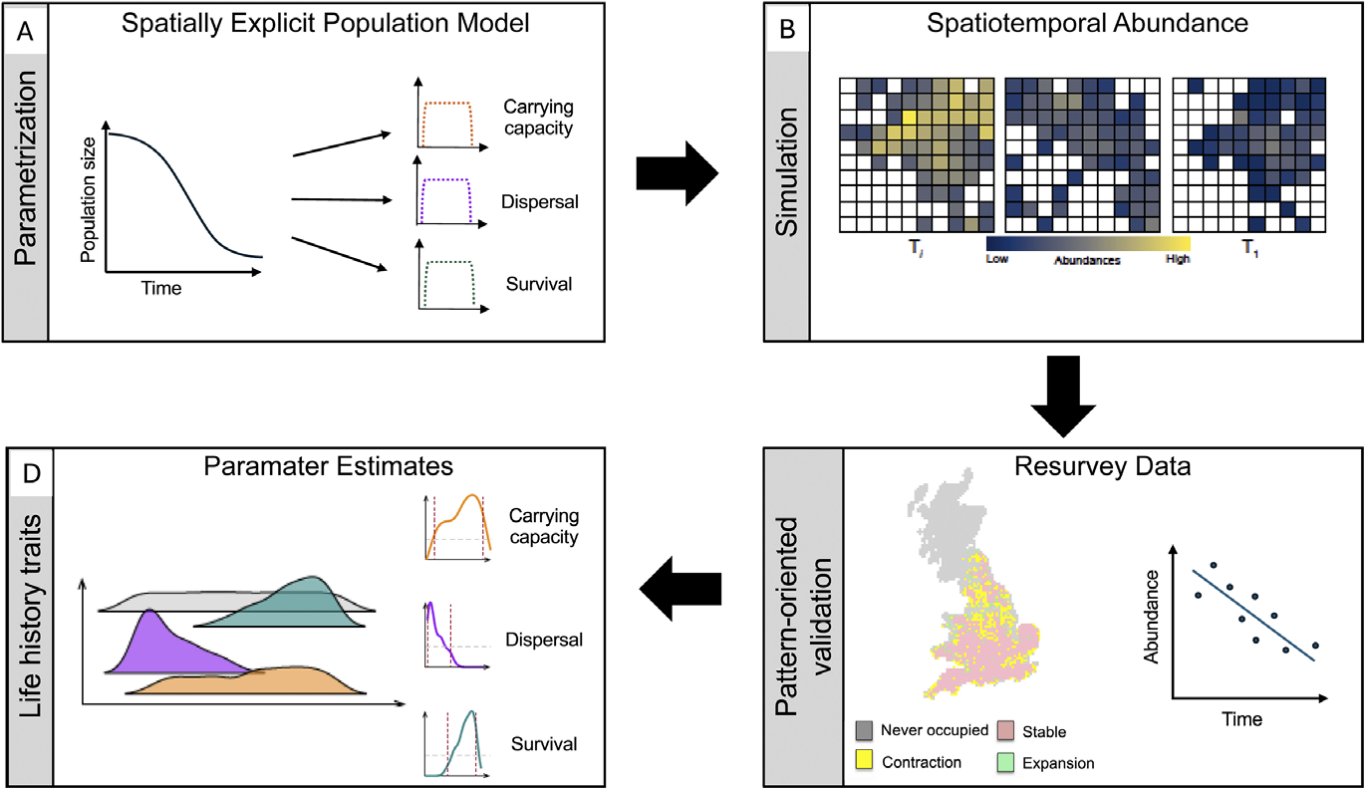
Identifying biological traits using process-explicit models, pattern-oriented validation, and resurvey data. A) Spatially explicit population models are built using life history parameters drawn from wide but plausible ranges. B) These models simulate thousands of plausible patterns of spatiotemporal abundance. C) Pattern-oriented modelling methods are used to validate these reconstructions of spatiotemporal abundance using observations of demographic change (shifts in range and abundance) from resurvey data. D) This identifies parameter bounds for life history traits and establishes their relative importance for reconstructing observed shifts in species distributions and abundances in response to climate and environmental disturbances.

Fortunately, the computer modelling platforms needed to unpack complex interactions between species traits and climate change now exist and are widely accessible (Bocedi et al., 2014a; Fordham et al., 2021; Kearney and Porter, 2020; Visintin et al., 2020). However, these process-driven modelling approaches continue to be primarily used to simulate the impact of forecast climate change on future species ranges and abundances (Briscoe et al., 2019). This is despite having capacities to directly identify species traits associated with demographic responses to observed climate change (Pilowsky et al., 2022a). Future research should now centre on using process-explicit models to analyse the growing volume of recent observations of spatiotemporal changes in species ranges and abundances in response to accelerating climate change (Lenoir et al., 2020; Rosenberg et al., 2019). Particularly, if these observed demographic changes are used as targets in process-explicit and pattern-oriented models (Figure 1). These established methods and data promise to provide a deeper understanding of the traits that species use to respond to climate and environmental disturbances, improving indicators of the future vulnerability of biodiversity to worsening planetary change. Because process-explicit modelling frameworks can be timely to construct, particularly when applied to multiple species, the initial focus could be on developing these process-driven approaches for select species that enable testing of climate-sensitive traits previously identified with statistical analysis of repeat survey data.

Longitudinal data on the abundances and ranges of a wide diversity of Earth’s plants and animals, living in terrestrial, marine and freshwater biomes, in open-access portals and databases has grown rapidly in recent decades (Edgar and Stuart-Smith, 2014; Sabatini et al., 2021). As has the diversity of species trait characteristics in online databases (Tobias et al., 2022; Wilman et al., 2014). As human-induced climate change worsens in the coming decades, these resources will become invaluable, particularly if they are regularly updated and expanded, enabling pronounced climate-driven responses of biodiversity to be followed in-situ. Analyses of these repeat survey data with process-driven modelling approaches and pattern-oriented methods will overcome many of the problems that have so far limited the identification of functional and life history traits associated with extinction proneness to climate change using correlative analytical methods. These include problems with analysing data that is sparsely distributed in space and time, and issues with disentangling complex non-linear trait-based responses to climatic change and their interactions with other anthropogenic threats.

Temporally and spatially explicit population models have already been used to reconstruct the range and population dynamics underpinning observed snapshots of trajectories of abundances and distributions of birds in the UK at fine temporal and spatial scales, and across large geographical extents (Fordham et al., 2018). Applying these and other process-explicit modelling methods to repeat survey data for species with diverse taxonomic coverage, is likely to reveal generalisable functional and life-history traits responsible for the fitness and persistence of species in shifting climates (Figure 1). These findings are needed immediately to strengthen the relevance of species traits in setting national and international conservation policy goals (Kissling et al., 2018).

Given that it is now possible to use process-explicit models to establish ecological and demographic determinants of species range shifts and population declines that occurred hundreds to thousands of years ago, more emphasis should also be placed on identifying biological responses to rapid climatic events that occurred in the more distant past (Pilowsky et al., 2022a). Times and places where paces and magnitudes of past and future forecasts closely match, and where sufficient paleontological data on biotic responses to these warming events exists, will provide important opportunities to better anticipate and manage the responses of species to changing climates (Fordham et al., 2020).

## Conclusion

Process-explicit models are providing a more complete understanding of the functional and life-history traits that regulate species’ responses to climate change. When run at fine temporal and spatial scales, and across large geographical extents, they can identify traits that make certain species particularly vulnerable to climate change. A greater emphasis on using process-driven modelling techniques to analyses the growing volume of data on past changes in species ranges and abundances is likely to better identify trait-based responses of species to climatic change, and strengthen conservation policies for protecting species that are most vulnerable to future climate warming.

## Data Availability

There is no empirical data supporting this paper.
