# Supplementary material for: Identifying species traits that predict vulnerability to climate change
Source: Camb Prism Extinct. 2024 Dec 5;2:e21. doi: 10.1017/ext.2024.24 (PMC11895733; doi:10.1017/ext.2024.24)
Supplement: Peer review history

# Editor report: Recommendation: Identifying species traits that predict vulnerability to climate change — R0/PR2

**DOI:** 10.1017/ext.2024.24.pr2

**Affiliations:** School of Natural Sciences, University of Tasmania, Australia

I am acting as EiC and HE due to unavailability of other editors.

# Editor report: Decision: Identifying species traits that predict vulnerability to climate change — R0/PR3

**DOI:** 10.1017/ext.2024.24.pr3

**Affiliations:** School of Natural Sciences, University of Tasmania, Australia

No accompanying comment.

# Editor report: Recommendation: Identifying species traits that predict vulnerability to climate change — R1/PR5

**DOI:** 10.1017/ext.2024.24.pr5

**Affiliations:** School of Natural Sciences, University of Tasmania, Australia

Acceptable after revising (minor revision) to satisfy referee critiques.

# Editor report: Decision: Identifying species traits that predict vulnerability to climate change — R1/PR6

**DOI:** 10.1017/ext.2024.24.pr6

**Affiliations:** School of Biological Sciences, University of Nebraska-Lincoln, United States

No accompanying comment.
